# Outcome of anticoagulation with rivaroxaban in patients with non-retrieved inferior vena cava filters for the prevention of filter thrombosis: a retrospective cohort study

**DOI:** 10.1186/s12872-022-02849-6

**Published:** 2022-09-11

**Authors:** Baoyan Wang, Chenxiao Jiang, Yepeng Zhang, Xiaoqiang Li, Hang Xu

**Affiliations:** 1 Department of Pharmacy, Nanjing Drum Tower Hospital, The Affiliated Hospital of Nanjing University Medical School, Nanjing, 210008 China; 2 Department of Vascular Surgery, Nanjing Drum Tower Hospital, The Affiliated Hospital of Nanjing University Medical School, Nanjing, 210008 China

**Keywords:** Venous thromboembolism, Inferior vena cava filter, Filter thrombosis, Rivaroxaban

## Abstract

**Background:**

Non-retrieved inferior vena cava filter (IVCF) is associated with some severe complications, such as filter thrombosis. The aim of this retrospective cohort study was to evaluate the outcome of rivaroxaban for the prevention of filter thrombosis in patients with non-retrieved IVCF.

**Methods:**

The study based on the VTE registry databases was limited to patients with non-retrieved IVCF treated at Nanjing Drum Tower Hospital from January 2012 to December 2017. Outcomes included filter thrombosis, total bleeding events, death.

**Results:**

A total of 202 patients were enrolled in the study and divided into rivaroxaban group and warfarin group. Mean follow-up period of the two groups was 57.4 ± 20.8 and 62.2 ± 23.0 months, respectively. In risk factors for VTE, transient factors (*P* = 0.008) and history of VTE (*P* = 0.028) were statistically different between the two groups. A total of 13 (6.4%) patients developed filter complications, of which 4 (3.5%) and 5 (5.7%) patients in rivaroxaban group and warfarin group developed filter thrombosis, respectively, without significant difference (*P* = 0.690). The total bleeding events in rivaroxaban group, including major bleeding and clinically relevant and non-major (CRNM) bleeding, were significantly lower than that in warfarin group (*P* = 0.005). Adjusting for hypertension, transient risk factors, history of VTE and cancer, no differences in the hazard ratio for outcomes were notable.

**Conclusions:**

It is necessary to perform a concomitant anticoagulation in patients with non-retrieved filters. Rivaroxaban can be an alternative anticoagulant option for the prevention of filter thrombosis.

## Introduction

Venous thromboembolism (VTE), including deep vein thrombosis (DVT) and pulmonary embolism (PE) is the important cause of mortality in hospitalized patients. Although anticoagulation is the first-line therapy for VTE, it is not suitable for certain patients with active hemorrhage, serious liver disease or coagulation defect [1]. Inferior vena cava filter (IVCF) has been demonstrated to be an alternative treatment option in the long-term prevention of PE. The current guidelines support that IVCF should be applied to prevent PE for patients who have contraindications to anticoagulation or have recurrent VTE with adequate anticoagulation [2].

In the past few decades, the insertion of IVCF has increased dramatically, including the use as an additional therapy to anticoagulation in patients with acute VTE or deep venous thrombosis prophylaxis during surgical procedure [3]. Considering the long-term safety of permanent IVCF, there has been interest in the application of retrievable IVCF. However, there are some special conditions in which the retrievable filters were not successfully retrievable. IVCF, providing long-term protection from potentially fatal PE, is associated with some severe complications, such as filter thrombosis, filter tilt, vena cava wall penetration, filter migration, filter fracture, recurrent VTE [4–6]. Filter thrombosis, a well-documented complication, has a less than 10% incidence rate with contemporary filters but published rates range widely from 2 to 30% [7]. Based on our observations, filter thrombosis could lead to inferior vena cava obstruction or recurrence of VTE in severe cases [8]. Currently, the paucity of clinical studies failed to provide positive recommendations on the optimal anticoagulation and duration following non-retrieved filters insertion.

Generally, vitamin K antagonists were prescribed with long-term administration for the prevention of recurrent VTE, with a target international normalized ratio (INR) of 2.0 to 3.0. Many published studies demonstrated that post-procedural anticoagulation significantly reduced the incidence of filter thrombosis or recurrent VTE, but with an increase in bleeding complications for long-term administration [9, 10]. To date, several non-vitamin K oral anticoagulants (NOACs) have been approved for prophylaxis or treatment of VTE. Rivaroxaban, an oral direct Factor Xa inhibitor, demonstrated non-inferiority compared to enoxaparin-vitamin K antagonist, but was associated with lower risk of major bleeding for acute VTE [11]. Moreover, standard and lower dose rivaroxaban could be considered for extended treatment of VTE, according to the EINSTEIN-CHOICE study [12]. Unfortunately, patients with IVCF insertion were not involved in those well-known clinical trials.

Nowadays, there have been few published studies relating to the concomitant anticoagulation of rivaroxaban following non-retrieved IVCF insertion. The objective of this retrospective cohort study was to investigate the outcome of rivaroxaban in patients with non-retrieved IVCF for the prevention of filter thrombosis.

## Methods

### Study design and patients

In this retrospective cohort study, consecutive patients with acute VTE were identified by reviewing the VTE registry databases in Nanjing Drum Tower Hospital from January 1, 2012 to December 31, 2017. The diagnosis of acute VTE was confirmed by using venography, Doppler ultrasonography or CT pulmonary angiography. Filters were inserted percutaneously via the femoral vein and located in the infrarenal inferior vena cava with the apex of the filters just below the level of the lowest renal vein. The patients with non-retrieved IVCF were included by reviewing the medical record and followed through the VTE Registry, as long as anticoagulation was still in use. Patients who did not receive anticoagulant therapy or had severe renal insufficiency (calculated creatinine clearance < 30 mL/min) were excluded from the analysis.

Anticoagulant therapy for patients with non-retrieved IVCF was carefully reviewed and recorded. Enrolled patients had been treated with an anticoagulant drug for three months after diagnosis of VTE, including a vitamin K antagonist or on-label dose of NOACs such as dabigatran, rivaroxaban or edoxaban. The patients with contraindication to anticoagulation due to active bleeding reinitiated anticoagulant therapy once bleedings had ceased. Then, patients were divided into two groups based on subsequent anticoagulant choice: (1) warfarin (maintaining INR at 2.0–3.0) or (2) NOAC. The NOAC group included only those patients treated with rivaroxaban, as other NOACs were minimally present in the population. The conventional dose of rivaroxaban for extended anticoagulant therapy is 10 mg daily.

### Data collection

Data were collected from the electronic medical record. Extracted data included demographic characteristics, inpatient and outpatient visit information, laboratory results, radiology images, and patient telephone. The demographic characteristics contained concomitant disease, risk factors for VTE, indication for IVCF insertion and filter type. The complications of filter were identified by reviewing the venography or venous Doppler ultrasonography results. The follow-up period began when the patients completed initial anticoagulant treatment for three months and lasted until the time of death or the end date of the study period (December 31, 2020). Incomplete follow-up information from electronic medical record was overcome with contacting patients by telephone.

### Study outcomes

The primary efficacy outcome was symptomatic filter thrombosis. The symptomatic filter thrombosis referred to reduced filter patency and venous return from the lower extremities, and could progress to inferior vena cava occlusion. According to the venous collateral formation and the extent of venous involvement, the patients had symptoms ranging from mild ambulatory lower extremity swelling to incapacitating edema at rest, venous claudication or venous ulcers. The symptomatic filter thrombosis had to be confirmed by venography or venous Doppler ultrasonography. The secondary outcome was death.

The safety outcomes included major bleeding and clinically relevant non-major (CRNM) bleeding. Major bleeding was defined as fatal bleeding, bleeding of a critical anatomic site, fall in hemoglobin concentration > 2 g/dL or transfusion of > 2 U of whole blood or packed red blood cells. CRNM bleeding was those that did not meet the criteria for major bleed, but led to medical intervention or cessation of drug treatment.

### Statistical analysis

Independent sample t-tests were used to analyze differences for continuous variables, expressed as mean ± standard deviation. Chi-squared tests or Fisher’s exact test were used for categorical data, expressed as frequency and percentages. The study outcomes between the rivaroxaban and warfarin groups were compared by Kaplan–Meier method and log-rank test for univariate analysis and time-dependent Cox proportional hazards models for multivariate analysis, which were reported as a hazard ratio (HR) with an associated 95% confidence interval (CI). Statistical analysis was performed with SPSS software (IBM Corporation, Armonk, NY). P-value < 0.05 was considered significant.

## Results

### Patient characteristics

**Fig. 1:**
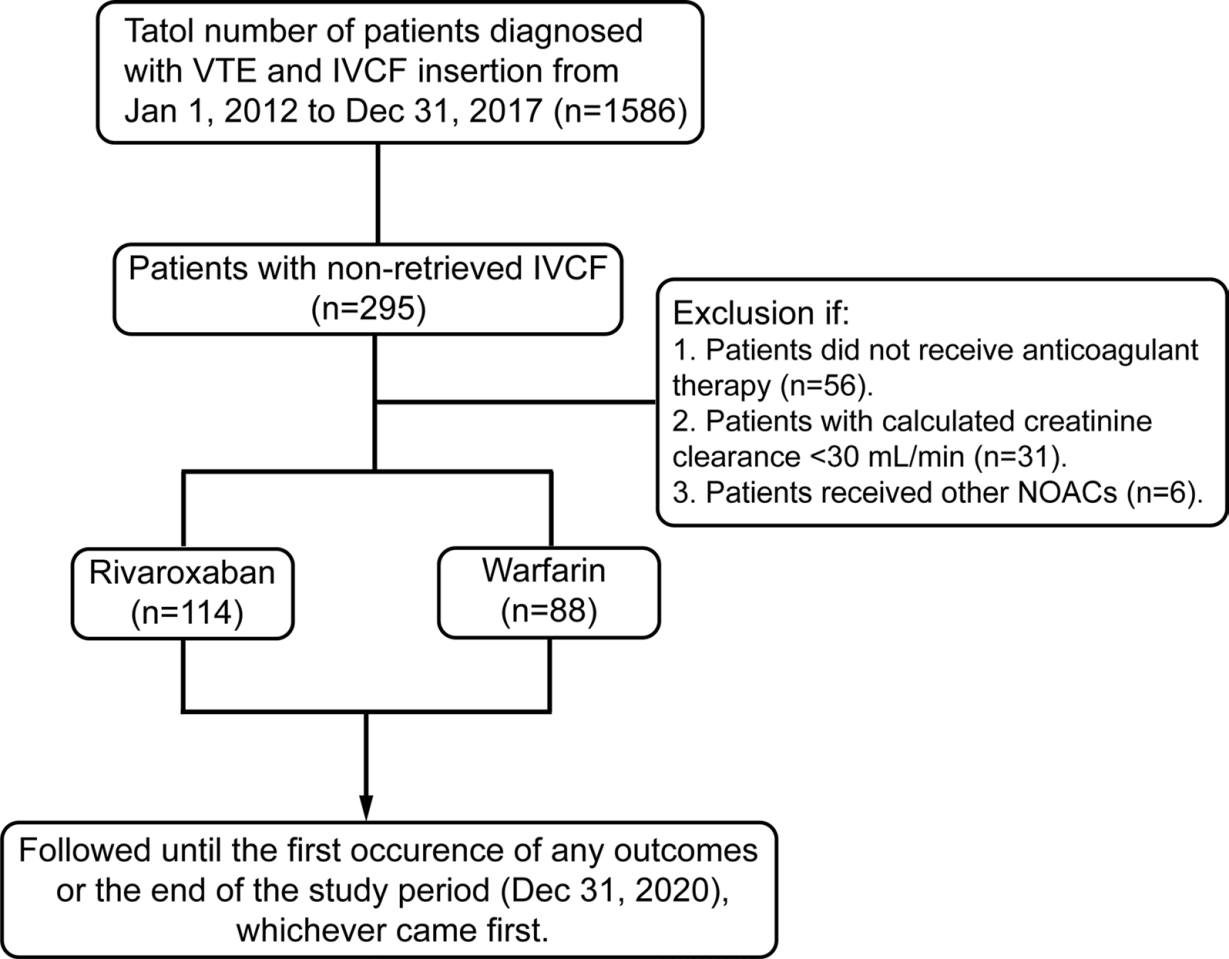
Flow diagram of patients identified, included and analyzed during the study period

Between January 2012 and December 2017, a total of 202 patients with the non-retrieved filters were included in this study (Fig. 1). Baseline characteristics of the patients were presented in Table 1. Patients with hypertension were more prevalent in rivaroxaban group (38.6% vs 23.9%, *P* = 0.026). Acquired transient risk factors, including major surgery, prolonged immobilization, bone fracture and puerperium, conveyed the most common risks for VTE, followed by cancer. There are more patients with transient risk factors in warfarin group than in rivaroxaban group (54.6% vs 36.0%, *P* = 0.008), as while the distribution of history of VTE was opposite (10.2% vs 21.9%, *P* = 0.028). The location of DVT in the two groups were mainly proximal. The most frequent indication for filter insertion was VTE with contraindications to anticoagulation in both groups. The contraindications to anticoagulation in this study referred to serious bleeding events, mainly including cerebral hemorrhage, gastrointestinal bleeding or other internal organs bleedings. Other indications included failure of anticoagulation, patients with acute VTE but required other surgical procedures, and insertion in the setting of catheter-directed thrombolysis. Of the filters which were inserted, the most commonly filter was OptEase and TrapEase filter. The OptEase filter, as well as the G2 and Aegisy filters, were all retrievable vena cava filters, which were not retrieved because of progressive contraindication to anticoagulation, large trapped thrombosis in the filter insertion site, failure to retrieve the filter and poor prognosis. Mean follow-up periods were 57.4 ± 20.8 months for rivaroxaban group and 62.2 ± 23.0 months for warfarin group, respectively, with no statistical difference.

**Table 1:** Baseline characteristics of the patients

	Rivaroxaban group	Warfarin group	*P* value
Number of subjects	114	88	
Mean age	60.1 ± 13.2	57.3 ± 12.6	0.130
Male/Female	65/49	54/34	0.534
Concomitant disease			
Hypertension	44(38.6%)	21(23.9%)	0.026
Diabetes	18(15.8%)	7(8.0%)	0.094
Coronary heart disease	10(8.8%)	6(6.8%)	0.610
Cerebral infarction	11(9.7%)	4(4.6%)	0.170
Risk factors for VTE			
Transient risk factors	41(36.0%)	48(54.6%)	0.008
History of VTE	25(21.9%)	9(10.2%)	0.028
Cancer	28(24.6%)	16(18.2%)	0.276
Thrombophilia	4(3.5%)	3(3.4%)	1.000
Type of VTE			
DVT only	98(86.0%)	82(93.2%)	0.103
DVT and PE	16(14.0%)	6(6.8%)	
Location of DVT			
Proximal	110(96.5%)	83(94.3%)	0.690
Distal	4(3.5%)	5(5.7%)	
Indication for IVCF insertion			
Contraindications to anticoagulation	46(40.3%)	34(38.7%)	0.476
VTE with failure of anticoagulation	14(12.3%)	6(6.8%)	
Perioperative operation	19(16.7%)	20(22.7%)	
Thrombolysis	35(30.7%)	28(31.8%)	
Filter type			
TrapEase filter (Cordis)	36(31.6%)	20(22.7%)	0.156
OptEase filter (Cordis)	38(33.3%)	34(38.6%)	
G2 filter (Bard)	16(14.0%)	7(8.0%%)	
Aegisy filter (China)	24(21.1%)	27(30.7%)	
Length of follow-up(months)	57.4 ± 20.8	62.2 ± 23.0	0.158

### Clinical outcomes

**Fig. 2:**
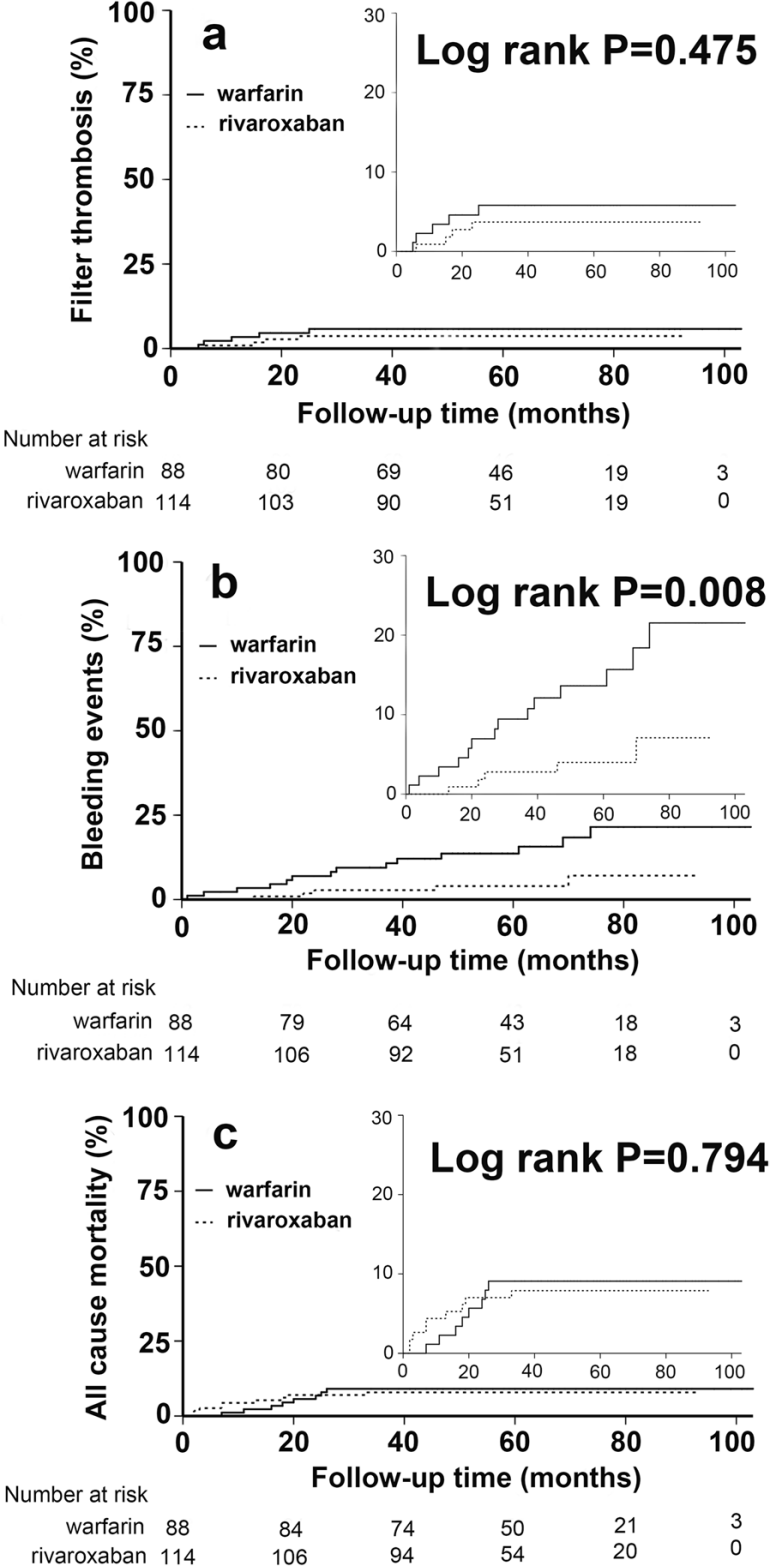
Kaplan–Meier analysis of filter thrombosis, bleeding events and all cause mortality

Table 2 listed the clinical outcomes after filter insertion. The filter thrombosis was recorded in 4 (3.5%) and 5 (5.7%) patients in rivaroxaban group and warfarin group during the follow-up period, respectively, with no significant difference (Fig. 2a, Table 2). The time to symptomatic filter thrombosis varied over a broad range of 147–748 days, most of which occurred within two years of filter insertion. The other complications, including filter tilt, vena cava wall penetration and filter migration occurred sporadically (Table 2). Recurrent VTE occurred in 2 patients in rivaroxaban group and 4 patients including one patient with fatal PE in warfarin group (1.8% vs 4.6%, *P* = 0.407). The composite safety outcomes of major bleeding and CRNM bleeding was lower in rivaroxaban group than that in warfarin group (Fig. 2b, Table 2). A total of three major bleeding events occurred in the two groups, including 1 case of gastrointestinal bleeding in the warfarin and rivaroxaban group, respectively, and 1 case of urinary tract bleeding in warfarin group. 4 patients in rivaroxaban group and 12 patients in warfarin group experienced CRNM bleeding events, respectively, with statistical difference (Table 2), including 7 cases of extensive skin bruising, 2 cases of gingival bleeding, 4 cases of fundus hemorrhage, 1 case of epistaxis, 1 case of increased menstruation bleeding, and 1 case of intramuscular hematoma. There was a total of 17 deaths in both groups with no statistically significant difference (Fig. 2c, Table 2) and most deaths were cancer-related. All deaths occurred within two years after filter insertion. Moreover, univariate and multivariate Cox proportional hazards models were undertaken to better understand the potentially confounding clinical variables contributing to clinical outcomes. Adjusting for hypertension, transient risk factors, history of VTE and cancer, no differences in the hazard ratio for outcome events including filter thrombosis, bleeding events, or all-cause mortality were notable (Table 3).

**Table 2:** Clinical outcomes after filter insertion

	Rivaroxaban group	Warfarin group	*P* value
Filter complications			
Symptomatic filter thrombosis	4(3.5%)	5(5.7%)	0.690
TrapEase filter	0	2	
OptEase filter	3	1	
G2 filter	0	1	
Aegisy filter	1	1	
Other complications	2(1.8%)	2(2.3%)	1.000
Time to symptomatic filter thrombosis			
Mean days (SD)	439.3(214.9)	369.8(245.3)	0.624
Median	462.0	308.0	
Q1, Q3	223.8, 632.0	164.5, 606.0	
Recurrent VTE			
Total	2(1.8%)	4(4.6%)	0.407
DVT	2(1.8%)	3(3.4%)	0.655
PE	–	1(1.1%)	
Deaths			
Total	9(7.9%)	8(9.1%)	0.761
VTE-related	0	1(1.1%)	
Cancer-related	5(4.4%)	4(4.5%)	1.000
Others	4(3.5%)	3(3.4%)	1.000
Safety outcomes			
Total	5(4.4%)	14(15.9%)	0.005
Major bleeding	1(0.9%)	2(2.3%)	0.581
CRNM bleeding	4(3.5%)	12(13.6%)	0.008

**Table 3:** Outcomes after adjustment for hypertension, transient risk factors, history of VTE and cancer

Outcomes	Unadjusted HR^a^ (95% CI)	*P* value	Adjusted HR^b^ (95% CI)	*P* value
Filter thrombosis	0.622 (0.167–2.318)^c^	0.480	0.459 (0.119–1.770)^c^	0.258
Bleeding events	0.276 (0.099–0.768)^c^	0.014	0.277 (0.096–0.803)^c^	0.018
All-cause mortality	0.881 (0.340–2.284)^c^	0.795	0.764 (0.281–2.078)^c^	0.598

^a^Unadjusted model only studies the relationship between the outcomes and anticoagulant drugs

^b^Adjusted model studies the relationship between the outcomes, anticoagulant drugs and adjustment factors

^c^warfarin was used as a reference

### Therapy for filter thrombosis

TrapEase filter (n = 2), OptEase filter (n = 4) and Aegisy filter (n = 2) with opposed biconical design was commonly associated with filter thrombosis (Table 2). It was worth noting that two patients in each group with filter thrombosis were diagnosed with cancer. The patients suffered thrombosis in inferior vena cava or iliac vein, leading to vascular stenosis or occlusion with the symptoms of pain or limb swelling. There were two patients in each group treated with catheter-directed thrombolysis, with successful recanalization of inferior vena cava, although it was unable to completely remove the thrombosis from the iliac vein. The other patents, presented with iliac vein occlusion and venous collateral circulation, underwent endovascular balloon expansion and stent implantation, with successful re-establishment of iliac vein blood flow.

### Time in therapeutic range with warfarin

The quality of warfarin therapy measured by time in therapeutic range (TTR) has been shown in Table 4. The evaluation criteria referred to a population-average model provided in the published articles. Most patients had a TTR range of 58% to 70%, followed by TTR below 58%, associated with even lower benefit from warfarin anticoagulation. The occurrence of complications for bleedings and thrombosis events was often concentrated in patients with lower levels of TTR. There was one major bleeding event occurred in patients with TTR below 58% and 58% ≤ TTR ≤ 70%, respectively. The INR at the time of major bleeding was 4.91 for the gastrointestinal bleeding and 2.59 for the urinary tract bleeding, no bleeding was fatal.

**Table 4:** Different levels of individual TTR

	TTR > 70%	58% ≤ TTR ≤ 70%	TTR < 58%
Number of patients	16	38	34
Bleeding events			
Major bleeding		1	1
CRNM bleeding	2	4	6
Thrombotic events			
VTE recurrence		3	1
Filter thrombosis			5

## Discussion

IVCF has been widely used in the daily clinical practice, although some studies have demonstrated that the application of retrievable IVCF did not prevent VTE recurrence or mortality compared with anticoagulation alone [6, 13]. In our medical center, the proportion of patients undergoing thrombolysis therapy accounted for a sizable part of patients with IVCF placement. Concerns regarding the long-term outcomes of filters have resulted in the application of retrievable IVCF, especially in patients with a long life expectancy. Actually, there were always some reasons why the filter cannot be retrieved from patient who no longer requires transient protection against PE.

To date, whether it is necessary to perform concomitant anticoagulation following non-retrieved IVCF insertion remains controversial. There are some published studies that advocate the application of post-filter anticoagulation therapy which reduced the occurrence of filter-related thrombosis or recurrent VTE and should be performed whenever possible [14, 15]. Conversely, several other studies evaluated the benefits of concomitant anticoagulation and found no evidence suggesting that such therapy was necessary [16, 17]. The incidence of VTE following filter insertion with concomitant anticoagulation was not statistically different from that in subjects who did not receive anticoagulation. Generally, the decision whether or not to perform concomitant anticoagulation depended on the preference of surgeon. There are many potential advantages of concomitant anticoagulation following filter insertion. Firstly, it may prevent the progress of thrombosis. Secondly, it may decrease the occurrence of filter thrombosis following insertion. Thirdly, it may further decrease the risk of recurrent VTE, especially in patients presenting with a first episode of unprovoked VTE. However, the adverse events of long-term anticoagulation include well-known risks of complications, particularly bleeding complications, in patients receiving anticoagulation. Balancing the competing risks of thrombosis and bleeding can be a difficult choice in these patients. In our study, probably due to the concomitant anticoagulation, the incidence of filter thrombosis was lower than that reported in the published studies [7].

The commonly available oral anticoagulation drugs in patients undergoing filter insertion were vitamin K antagonists which inhibit the synthesis of vitamin K-dependent coagulation factors. However, whether or not the patients receiving adequate anticoagulation or the duration was not presented in most studies. Currently, insufficient evidence exists to establish a standardized warfarin anticoagulation protocol for the prevention of filter thrombosis and recurrent VTE after filter insertion. In our medical center, some patients still chose warfarin as long-term anticoagulant therapy option. Unfortunately, severe complications or patient compliance with regular INR testing leaded to the cessation of warfarin administration. As for rivaroxaban, the EINSTEIN-DVT study demonstrated that rivaroxaban was not inferior to warfarin in the treatment of VTE. However, the study was based on the general VTE population and patients with IVCF insertion were excluded. Whether or not rivaroxaban is effective anticoagulant option for filter thrombosis is uncertain. In our study, the extended anticoagulant therapy was rivaroxaban 10 mg daily and the results showed that rivaroxaban did not reduce the incidence of filter thrombosis, but it could significantly reduce the incidence of bleeding events. This may be due to the fact that patients taking warfarin were unable to regularly monitor blood coagulation for long time. Unlike warfarin, rivaroxaban has clinical advantages of fixed dosage, require no coagulation monitoring and fewer drug-drug interaction. Considering the necessity of long-term anticoagulation for patients with non-retrieved IVCF, rivaroxaban should be a reasonable and valuable option.

According to our study, the observed difference in thrombosis incidence may be attributed to inherent filter design differences. Most symptomatic filter thrombosis occurred in the patients with TrapEase, OptEase or Aegisy IVC filters, which had the opposed biconical design. The structural style has been reported to have a higher incidence of filter thrombosis [18, 19]. Based on a vitro study investigating the hemodynamic effects of thrombosis entrapment by the TrapEase filter [20], the opposed biconical design made the thrombosis to be trapped between the filter and the vessel wall in the inferior region. The margination effect would generate a large region of flow stagnation that is considered to be the potential mechanism of flow-induced filter thrombosis. Alternatively, the design was intended to capture migrating thrombosis more effectively and limit the migration of filter. This margination effect did not appear in the single-cone design of the Greenfield, Günther Tulip and G2 filters [21, 22], which trapped the thrombosis to a central location where the blood flow was relatively high.

Moreover, two patients in each group with filter thrombosis were diagnosed with cancer, including lung adenocarcinoma, gastric cancer, ovarian cancer and pelvic cancer. It is well known that the hypercoagulable state has been recognized in patients with cancer. Patients with active cancer or those undergoing active treatment frequently experience thrombosis events [23]. Rivaroxaban 10 mg daily may indicate insufficient anticoagulant intensity for the prevention of filter thrombosis in those patients. These appears to be a need for further research to determine the optimal dose of extended thrombosis prophylaxis. Due to the high risks for VTE recurrence, patients with cancer might often be considered for IVCF placement in the real world. Therefore, the optimal long-term anticoagulation of these patients should be enough to arouse our attention.

This study also has several limitations. Firstly, the study was a retrospective cohort study, which can be influenced by the selection bias or unmeasured confounders. The filter placement and choice of filter type were determined by the physician. Secondly, although the time span was extended, the sample size in the study was insufficient because of few patients who need permanent IVCF insertion and low filter recovery failure rate in our urban medical center. Therefore, further and larger studies are needed to draw definite conclusions.

## Conclusions

Non-retrieved inferior vena cava filters could increase the risk of filter thrombosis. Although there have been no prospective randomized controlled trials to evaluate the efficacy and safety of rivaroxaban in the prevention of filter thrombosis, rivaroxaban can be a valuable option for long-term anticoagulant therapy.

## Data Availability

The datasets generated during the current study are available from the corresponding author on reasonable request.

## Abbreviations

IVCF: Inferior vena cava filter
VTE: Venous thromboembolism
DVT: Deep vein thrombosis
PE: Pulmonary embolism
NOACs: Non-vitamin K oral anticoagulants
CRNM: Clinically relevant and non-major
HR: Hazard ratio
CI: Confidence interval
